# Theoretical evaluation of the performance of IRMOFs and M-MOF-74 in the formation of 5-fluorouracil@MOF

**DOI:** 10.1039/d1ra05068k

**Published:** 2021-09-20

**Authors:** Nailton M. Rodrigues, João B. L. Martins

**Affiliations:** Instituto de Química, Universidade de Brasília Brasília – DF 70910-900 Brazil nailton.rodrigues@unb.br

## Abstract

Drug delivery systems are a viable resource to be used in medical treatments that tend to be very aggressive to patients, increasing the bioavailability. In this context, porous structures such as MOFs emerge as promising for this type of application, in which a specific drug is adsorbed onto the structure for further release. MOFs such as IRMOFs and M-MOF-74 are investigated in many applications, including use as a drug carrier. In this work, the Monte Carlo grand canonical simulation was used for obtaining insights on the behaviour of 5-fluorouracil adsorption on IRMOF-1, IRMOF-8, IRMOF-10, Mg-MOF74, Fe-MOF74, Cu-MOF74 and Zn-MOF74. We have evaluated the influence of the adsorption of changing organic and inorganic units, which resulted in different chemical environments. It was seen that the drug interacts more efficiently with M-MOF-74, where the metallic centre plays an important role. For IRMOFs, a larger pore volume increases the amount of adsorbed molecules. This effect is mainly due to the contribution of the efficient interaction between 5-fluorouracil molecules.

## Abstract 2

Drug delivery systems are a viable resource to be used in medical treatments that tend to be very aggressive to patients, increasing the bioavailability.

## Graphical abstract

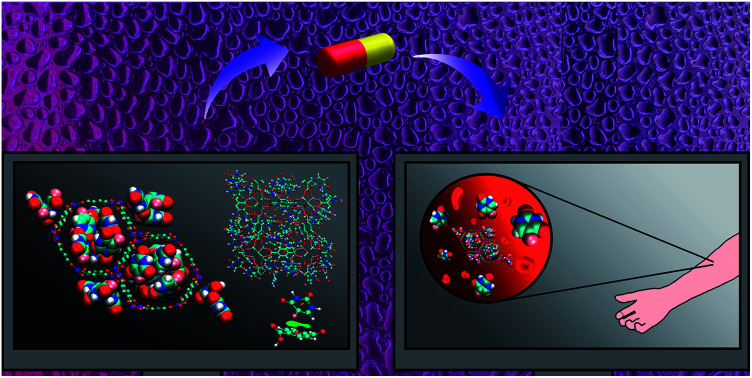

## 1. Introduction

Metal–organic frameworks (MOFs) are porous structures^1^ with applications in different areas.^2–4^ Among the known MOFs, one of the most investigated is MOF-5, which is also the precursor to Iso Reticular MOFs (IRMOFs).^5,6^ These new structures differ from each other only with regard to their organic unit, containing the same metallic centre in the inorganic unit, *i.e.*, zinc.^7^

Another family widely studied is M-MOF-74-X (M referring to the metallic centre^8^ and X referring to the organic unit used^9^), which have several hexagonal pore sizes.^10^ Among the various structures of this family, a group that stands out in the scientific community is the M-MOF-74-I (I is the 2,5-dihydroxybenzene-1,4-dicarboxylate (DBDC)).^11^ with a pore size of 10 to 12 Å as a microporous material.^12^ All members of that family as well as the IRMOFs, have interpenetrating structures,^13^ that is, they allow the entry of diverse molecules in their pores; together with their regular and permanent porosity, and high thermal stability (up to about 300 °C),^14^ several applications are possible.^15,16^ These structures are used for drug delivery^17^ and have been studied to form the drug-MOF system (drug@MOF).^18^ This system has the advantage of promoting the continuous and controlled release of the drug in the plasma.^19^ This possibility can impact the side effects of using drug concentrations^20^ exceeding the therapeutic range.^21,22^ Therefore, drug delivery studies^23^ are important,^24^ and contributions to this area are a challenge for improvement and development.^25^ Moreover, MOFs are generally reported to have good biocompatibility and are non-toxic to the human body.^26^ MOFs as Zn-MOFs and other systems can accumulate in the body and spleen and degrade into their constituent but are ultimately excreted in urine and faeces without any metabolization.^27^

5-Fluorouracil (5FU) is one of the drugs used in chemotherapy treatments for tumours^28^ such as oesophageal, stomach, ovarian, pancreatic and breast.^29^ The studies focusing on MOF as drug delivery for 5FU^30,31^ have been carried out by experimental^32–34^ and theoretical techniques.^35–37^ Using experimentation combined with computer simulations, Yan *et al.* studied the use of a Zn-MOF ([Zn_3_(OH)_2_(H_2_tccp)_2_(bpy)_2_](H_2_O)_3_(DMF)_3_) as drug delivery for 5FU.^38^ They showed that the MOF pore capture capacity was 30.7% with a controlled release in artificial plasma solution. The system was chosen as an efficient inhibitor of ovarian cancer cell proliferation from *in vitro* tests and *in vivo* model.^38^

Grand Canonical Monte Carlo simulation (GCMC)^39^ has been widely used in the theoretical study of adsorption on MOF systems.^40^ In this context, the RASPA program^41^ has been gaining prominence for having the GenericMOF force field, which is highly indicated to treat these structures.^42^ This is the case in the adsorption study carried out by Proenza and Longo, when applying the GenericMOF force field to describe the interactions between ZIF-8 and 5FU.^35^

In the MOF application as a drug carrier, the stability in the physiological environment and the toxicity of MOF in the humans should be highlighted.^43^ Moreover, the toxicity depends on factors related to the topology, pore, organic linkers and metal used in inorganic unit. Thus, MOFs in which these properties have already been investigated will be used in this work.

Finally, studies that lead to contributions of drug behaviour in the pore of MOFs, focusing on insights that enable its use in drug delivery,^44^ are of great importance. Semiempirical simulations were carried out to understand the behaviour of 5FU molecules when adsorbing in the pore of IRMOF-1, IRMOF-8, IRMOF-10, Mg-MOF74, Fe-MOF74, Cu-MOF74 and Zn-MOF74. The influence of different organic and inorganic units will be evaluated, which result in different chemical environments.

## 2. Methodology

The crystallographic structures for IRMOF-1, IRMOF-8 and IRMOF-10, were obtained in the CCDC (Cambridge Crystallographic Data Centre), and the Mg-MOF74, Fe-MOF74, Cu-MOF74 and Zn-MOF74 structures were obtained from the RASPA program database.^41^

**Fig. 1:**
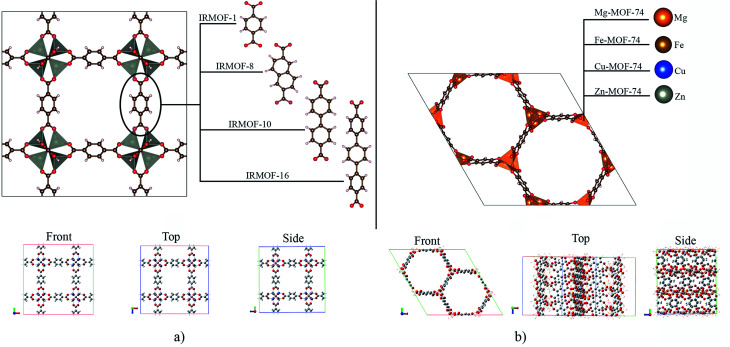
Structural representation of MOFs studied in this work, with: (a) IRMOFs and (b) M-MOF-74. Atom colour label: oxygen: red, carbon: brown, hydrogen: white, magnesium: orange, iron: gold, copper: blue, and zinc: grey.

IRMOFs have the same inorganic unit and different organic linkers, and their pore volumes are different (Fig. 1a). On the other hand, the M-MOF-74 structures have the same organic unit and different metal centres in the inorganic unit (Fig. 1b), not changing the pore volume. Therefore, Table 1 shows only one volume value for all the M-MOF-74 structures. The unit cell volume was calculated from *a*, *b* and *c* cell parameters of each MOF. The helium void fraction was calculated using the RASPA2.0 program.^41^ Taking in account this data, the pore volume was calculated by multiplying the unit cell volume by the void helium fraction.^41,45^

**Table 1:** Lattice parameters and pore volume for the different MOFs evaluated

MOF	Lattice parameters						Pore volume (Å^3^)
	*a*	*b*	*c*	*α*	*β*	*γ*	
IRMOF-1	23.8	23.8	23.8	90	90	90	15 184.39
IRMOF-8	30.1	30.1	30.1	90	90	90	22 394.38
IRMOF-10	34.3	34.3	34.3	90	90	90	34 748.03
M-MOF-74	26.1	26.1	20.8	90	90	120	1239.65

For each MOF, 5FU molecule adsorption was obtained with GCMC simulation using the RASPA program at 1.0 bar of pressure and 298 K of temperature. The calculation was carried out with 10 000 cycles of initialization and 30 000 cycles of simulation and a time step of 0.5 ms. The force field parameters used for all structures is contained in the Table 2.

**Table 2:** Lennard–Jones parameters for all atoms of the structures used in the GCMC simulations

Structure	Atom	*σ* (Å)	*ε*/*k*_b_ (K)
5FU^49^	C	3.340	43.292
	N	3.250	85.578
	O	3.340	105.724
	F	3.118	30.707
	H	1.069	7.903
IRMOFs^41^	Zn	2.462	62.399
	O_cen	3.033	48.158
	O_CO_2_	3.050	79.000
	C_CO_2_	2.800	27.000
	C_CH	3.600	30.700
	H	2.360	25.450
M-MOF-74 (ref. 41)	Mg	2.691	55.857
	Fe	2.594	6.542
	Cu	2.516	3.114
	Zn	2.462	62.399
	O_CO_2_	2.691	55.857
	O_CO	3.033	60.158
	C_CO_2_	2.800	27.000
	C_ben	3.617	74.856
	C_CH	3.875	19.632
	H	2.846	7.649

In the GCMC simulations, the number of molecules is determined by the system conditions (GC) and existing interactions (force field). At the same time, the starting position is defined by the random distribution (MC) and for the system energy. For GCMC simulations, the volume (*V*), temperature (*T*) and chemical potential (*μ*) are kept fixed (*μVT*). 5FU molecules can enter and exit the simulation box that is in equilibrium with the neighbour, which is a large reservoir of 5FU molecules.^46–48^ The simulation box for the IRMOFs was the 1 × 1 × 1 unit cell, and for the M-MOF-74 *a* 1 × 1 × 3 super cell was used. Box parameters can be seen in Table 1.

**Fig. 2:**
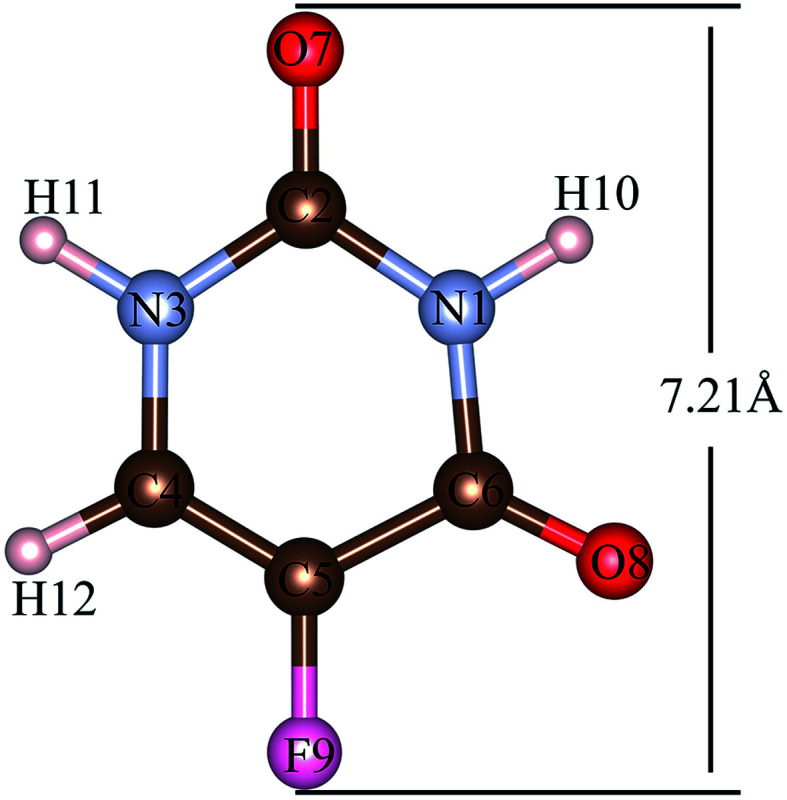
5FU structure with atom number label. Atom colour label: oxygen: red, nitrogen: blue, carbon: brown, fluorine: pink and hydrogen: white.

The 5FU (Fig. 2) is a considerably small molecule. The largest dimension of the 5FU is 7.21 Å, considering van der Waals radii, which yields to have free access to the pore of all evaluated MOFs for 5FU.

The atomic charges of 5FU were obtained using ChelpG atomic charges at B3LYP/6-311++G** level in the Gaussian09 program, revision D.01.^50^ For the MOF, the atomic charges were obtained from the charge-equilibration scheme of Wilmer *et al.*^51^ on the RASPA program, where the Ewald method^52,53^ was used for calculating the coulombic interaction. From these simulations, interaction energies and the energy from van der Waals and Coulomb contributions were obtained.

The equilibrium structure of the 5FU molecule in the drug@MOF system was investigated using PM6-D3,^54,55^ to calculate the interaction energy. PM6-D3 includes the Grimme dispersion correction. The PM6-D3 equilibrium structure was used as input in the Non-Covalent Interaction (NCI) simulations. The semiempirical calculation was performed using the MOPAC2016 program,^56^ and the NCI analysis was carried out in the NCIPLOT package.^57^

Due to the large size of these systems, including transition metals, the interaction energy between MOF and a 5FU molecule, when it interacts in the region of the inorganic unit, was obtained with B3LYP-D3/6-31G using the ORCA 4.2.1 program.^58^

**Fig. 3:**
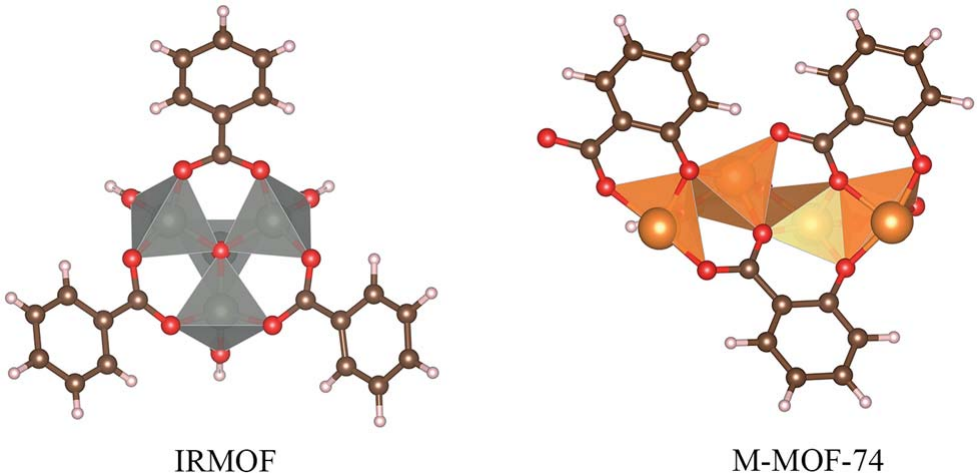
Cluster model used for interaction energy calculation using DFT-D3.

The calculation of DFT-D3 interaction energy was possible using a small cluster (Fig. 3) of 5FU@MOF isolated from GCMC results (MOF as cluster model).^59–62^ It is expected differences of the interaction energy obtained from cluster single point calculation compared to the periodic method. Furthermore, the magnetic ground state was calculated for Fe-MOF-74 and Cu-MOF-74 high spin states, multiplicity 5 for Fe-MOF-74 and 3 for Cu-MOF-74.

## 3. Results and discussion

Table 3 shows the 5FU adsorption data on the different MOFs. As expected, it is possible to observe for the IRMOFs that the adsorption increased with the increase in the pore volume (Table 3). Moreover, M-MOF-74 presents different adsorption values, which suggests the influence of each metal centre, with Zn-MOF-74 being the smallest adsorption, while Mg-MOF-74 has provided the highest adsorption capacity.

**Table 3:** 5FU adsorption capacity on different MOFs obtained at 1 bar and 298 K

MOF	Adsorption (mg g^−1^)
IRMOF-1	1428.14
IRMOF-8	2131.48
IRMOF-10	2839.74
Mg-MOF-74	777.50
Fe-MOF-74	594.34
Cu-MOF-74	602.17
Zn-MOF-74	548.62

**Fig. 4:**
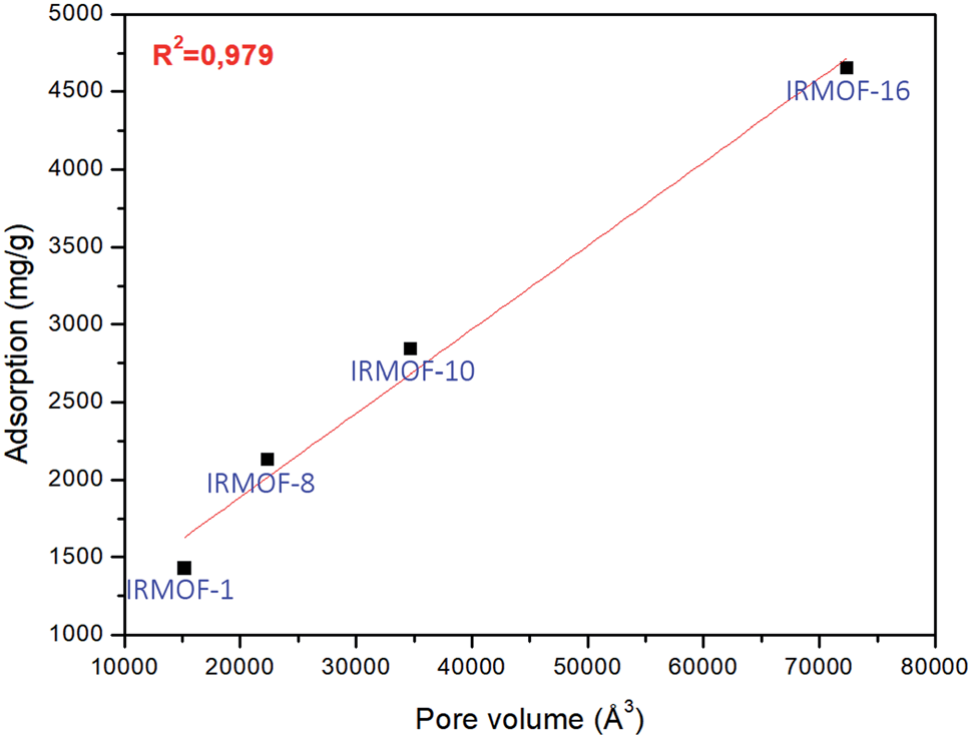
Linear regression for the relationship between pore volume and adsorption for 5FU on the different MOFs available.

The pore volume of the IRMOFs against adsorption values gives an almost linear trend, which indicates that IRMOFs with larger pores carry out more significant amounts of adsorbate, and the pore volume becomes the relevant factor in the process. This behaviour was also observed by Erucar and Keskin when evaluating 5FU adsorption on MOFs with different pore volumes.^63^ To complement this discussion, the adsorption of 5FU on IRMOF-16 was calculated. The pore volume of this structure is 72 403.77 Å^3^, and the calculated adsorption was 4648.79 mg g^−1^. For the data of four IRMOFs, the *R*^2^ = 0.979 was obtained (Fig. 4). Therefore, it is possible to suggest a direct relationship between IRMOF pore volume and adsorption (see Fig. 4). Pore volume increases as larger organic spacers are used, and these organic units are very similar chemically. They did not have significant changes in the chemical environment of the IRMOFs. Larger organic spacers explain that the increase in pore volume promotes a greater amount of 5FU adsorption with a linear relationship.

**Fig. 5:**
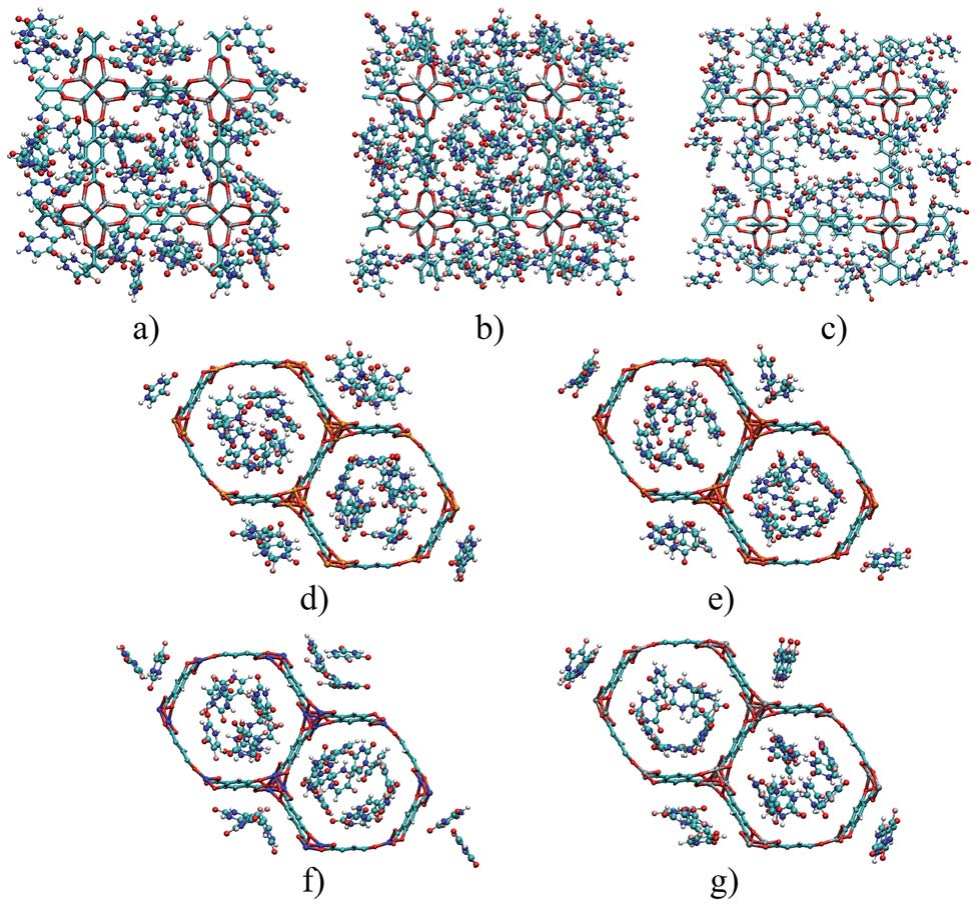
Distribution of the drug in different structures: (a) IRMOF-1, (b) IRMOF-8, (c) IRMOF-10, (d) Mg-MOF-74, (e) Fe-MOF-74, (f) Cu-MOF-74, and (g) Zn-MOF-74. Atom colour label: oxygen: red, nitrogen: blue, carbon: brown, hydrogen: white, magnesium: orange, iron: gold, copper: blue, and fluorine: pink.

Fig. 5 depicts the 5FU distribution on the studied MOFs. When analysing the results for IRMOF-1, IRMOF-8 and IRMOF-10, it is possible to observe that the 5FU molecules interact in various ways with the MOFs and among themselves.^64^ For this reason, they tend to occupy the entire pore volume (Fig. 5). For M-MOF-74, the 5FU also occupies the entire volume of these structures.

**Fig. 6:**
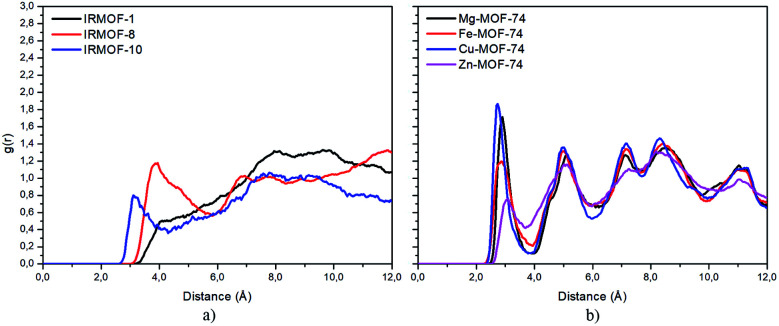
RDF of 5FU oxygen (O7) and metal atom from different MOFs.

Radial distribution function (RDF) is useful to discuss the distribution of 5FU molecules inside the pores. Fig. 6 shows the distribution function in relation to the interaction between the O7 of 5FU molecules (see Fig. 2) and the inorganic unit. The radial distribution (Fig. 6) shows that the minimum interaction distance tends to decrease with pore growth. In contrast, the interaction with the metal is more significant, which is evidenced by the peaks around 3.5 and 4.0 Å for IRMOF-8 and IRMOF-10, respectively. The IRMOF-1 presents a different behaviour. In the case of M-MOF-74, the interaction with Mg, Cu, and Fe occurs more frequently at shorter distances than for the Zn. For M-MOF-74 the first coordination sphere ends around 3.8 Å. Moreover, it is possible to note that the interactions between 5FU molecules and the inorganic unit are the most common, in agreement with the literature.^65^ However, being of lower frequency in Zn-MOF-74.

**Fig. 7:**
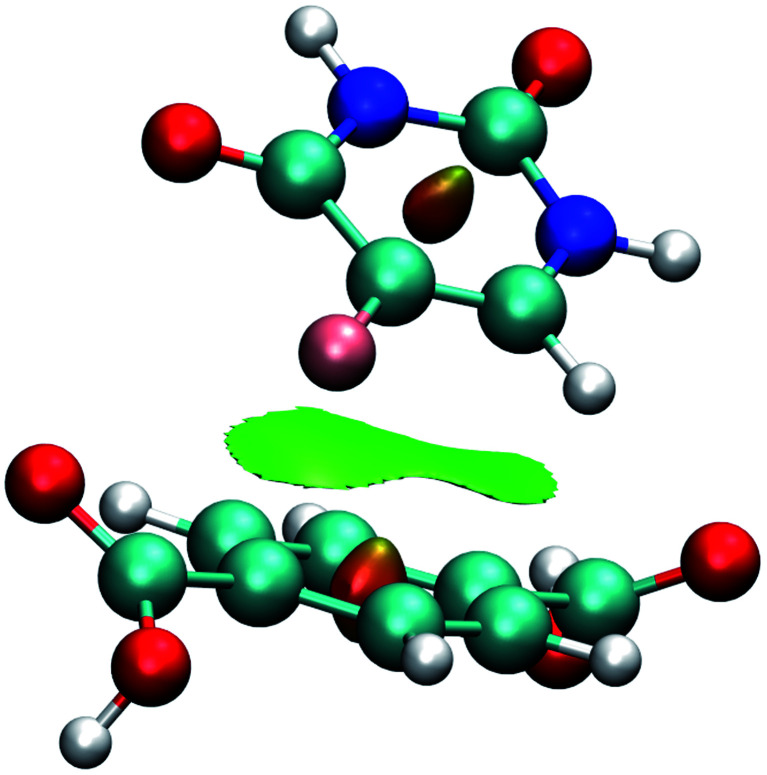
Interaction between a 5FU molecule and BDC ligand (IRMOF-1) from NCI of MCGC structure. Atom colour label: oxygen: red, nitrogen: blue, carbon: green, hydrogen: white and fluorine: pink.

Fig. 7 shows the interaction using IRMOF-1 structure with the organic unit of IRMOFs. The same profile is valid for the other structures. The 5FU molecules were found with interaction parallel to the aromatic ring of the BDC (1,4-benzodicarboxylate), where the ligand shows a π stacking interaction (Fig. 7). These observations indicate that the force field used is adequate to describe these interactions. Furthermore, through NCI calculations, it was seen that this interaction presents a green-coloured region between the rings. This is a weak interaction and agrees to the value found for the interaction energy calculated at PM6-D3 single point energy, yielding a value of 3.68 kcal mol^−1^. The interaction energy for PM6 without D3 correction is 0.36 kcal mol^−1^, showing the importance of the D3 function.

As previously stated in Fig. 5, many interactions occur between the 5FU and the MOFs and between the 5FU molecules. All interactions have different orientations and associated energy values, and for an isolated molecule of 5FU interacting in regions close to the inorganic unit of IRMOFs, these structures have higher interaction energy in relation to those close to the organic unit. In terms of percentage, it is approximately 55.34% higher in IRMOF-1, 42.75% in IRMOF-8 and 45.86% in IRMOF-10 (obtained with PM6-D3). The M-MOF-74 structure presents a more complex situation. This analysis was not possible due to 5FU interacting with more than one pore region in all cases of M-MOF-74.

**Fig. 8:**
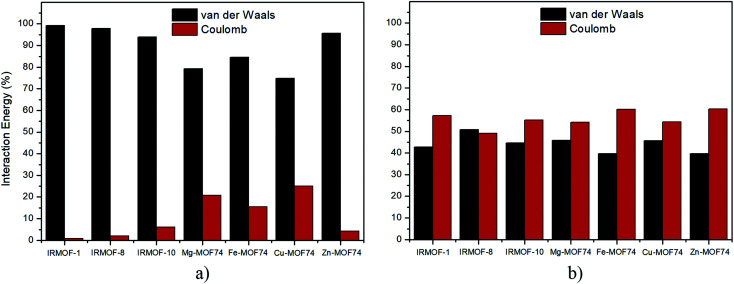
Percentage of contribution between van der Waals and Coulomb in the interactions: (a) 5FU-MOF, (b) 5FU–5FU.

Regarding the nature of 5FU-MOFs (Fig. 8a) and 5FU-5FU interactions (Fig. 8b), it was possible to address the components of van der Waals and Coulomb contributions for these interactions. IRMOFs present a major contribution for the 5FU-MOF interaction from van der Waals, which is expected since these are dependent on the pore size. Otherwise, the 5FU-MOF interaction of M-MOF-74 structures presents a significant electrostatic contribution since these structures present other metals than zinc, highlighting the importance of the metal present in this unit. Zn-MOF-74 shows the smallest Coulomb contribution of this group. Conversely, the 5FU–5FU interaction has almost the same contribution from van der Waals and Coulomb. Based on the results, it is possible to note that 5FU interacts with IRMOFs through predominantly weak interactions, so these interactions are easily broken. For M-MOF-74, the significant contribution by Coulomb forces tends to make the broken of these interactions more difficult.

**Fig. 9:**
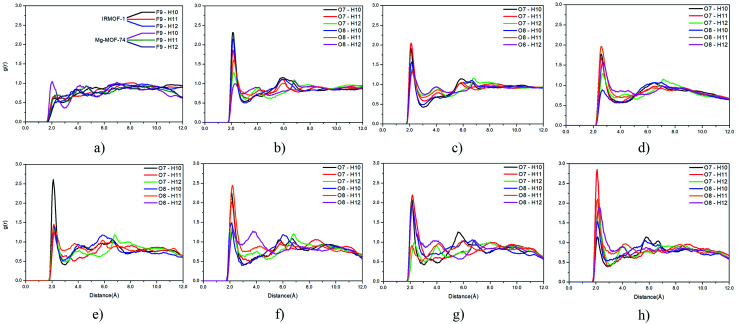
RDF of 5FU atoms: (a) F9, H10, H11 and H12 5FU atoms in the IRMOF-1 and Mg-MOF-74 pore, (b) O7, O8, H10, H11 and H12 in the IRMOF-1 pore, (c) O7, O8, H10, H11 and H12 5FU atoms in the IRMOF-8 pore, (d) O7, O8, H10, H11 and H12 5FU atoms in the IRMOF-10 pore, (e) O7, O8, H10, H11 and H12 5FU atoms in the Mg-MOF-74 pore, (f) O7, O8, H10, H11 and H12 5FU atoms in the Fe-MOF-74 pore, (g) O7, O8, H10, H11 and H12 5FU atoms in the Cu-MOF-74 pore, (h) O7, O8, H10, H11 and H12 5FU atoms in the Zn-MOF-74 pore.

Regarding the interactions between 5FU molecules (Fig. 9), the RDF provided important data on the main interactions between their atoms. The fluorine and hydrogen interactions (Fig. 9a) are less frequent than interactions between oxygen and hydrogen atoms (Fig. 9b–h). The distribution profile for interactions involving fluorine atoms within the different pores show negligible variation, and their results can be summarized in Fig. 9a.

For interactions between oxygen and hydrogen, the change is more accentuated. For interactions between O7, O8, and H10, H11 and H12, it was possible to verify that the chemical environment of the pore can induce restricted interactions. This trend is found when comparing the pore distribution profile of Mg-MOF-74 (Fig. 9e) and Zn-MOF-74 (Fig. 9h). O7 and H10 is the most frequent interaction in the Mg-MOF-74 pore, while the O7 with H11 interaction prevails in the Zn-MOF-74 pore, and the interaction between O7 and H10 becomes less frequent.

The average energies of all interactions obtained using the RASPA program are shown in Table 4. These results showed that 5FU molecules interact stronger with M-MOF-74 when compared with the IRMOFs. For the M-MOF-74 structure, the lowest interaction energy was found for Zn-MOF-74 (11.38 kcal mol^−1^) and the highest with Cu-MOF-74 (13.09 kcal mol^−1^). For IRMOFs, as the pore volume grows, the average interaction energy decreases, and this may be associated with an increasing number of interactions with the organic unit, which has weaker interaction and tends to reduce the average energy value. The average energy of interaction between 5FU molecules is greater than the energy obtained in interactions with IRMOFs, and less than that obtained for interactions with M-MOF-74, suggesting that interactions with IRMOFs are less effective than with M-MOF-74.

**Table 4:** Average interaction energy between 5FU-MOF and between 5FU molecules, obtained from GCMC simulations

MOF	Interaction energy (kcal mol^−1^)	
	5FU-MOF	5FU–5FU
IRMOF-1	5.97	14.13
IRMOF-8	4.99	14.20
IRMOF-10	3.40	12.44
Mg-MOF-74	12.90	11.11
Fe-MOF-74	12.08	11.21
Cu-MOF-74	13.09	11.36
Zn-MOF-74	10.89	11.38

**Fig. 10:**
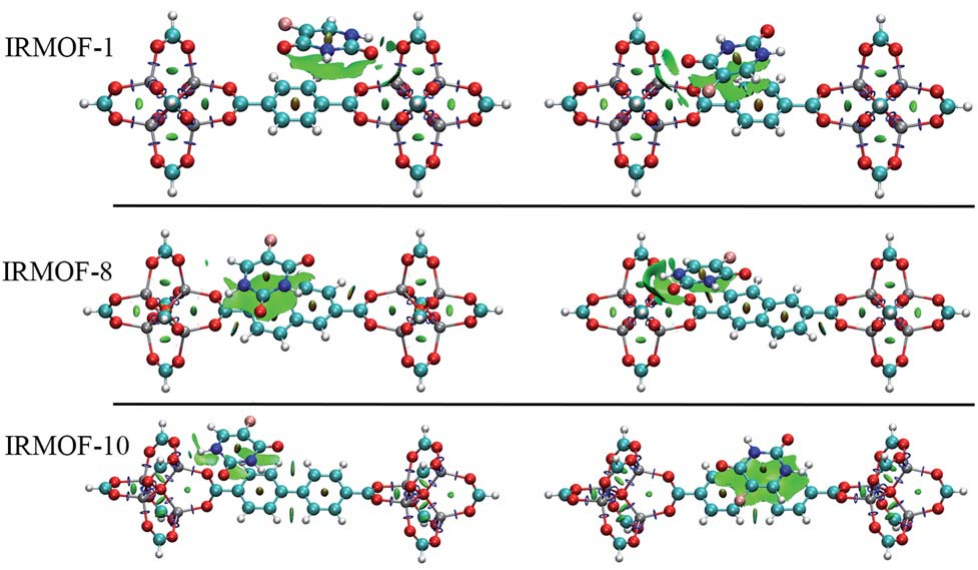
Minimum energy points for the interaction between 5FU and IRMOF-1, IRMOF-8 and IRMOF-10. Atom colour label: zinc: grey, oxygen: red, nitrogen: blue, carbon: green, hydrogen: white and fluorine: pink.

**Fig. 11:**
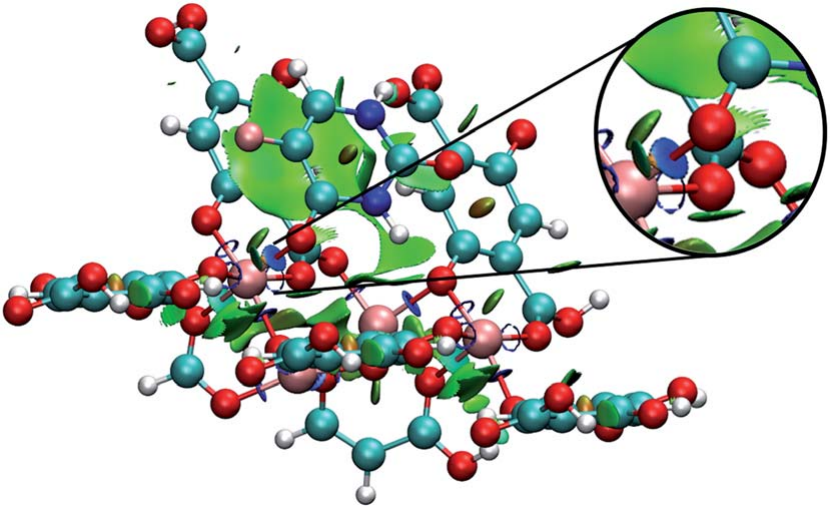
Minimum energy point for the interaction between a 5FU molecule and Fe-MOF-74. Highlighting the interaction between 5FU oxygen and an iron atom (in blue). Atom colour label: zinc: grey, iron: gold, oxygen: red, nitrogen: blue, carbon: green, hydrogen: white and fluorine: pink.

Analysing different minimum points through NCI calculations (Fig. 10 and 11), it was possible to identify the major contribution of weak interactions (in green) in the IRMOFs, and for M-MOF-74 it was observed the presence of stronger interactions (in blue). This is present in Cu-MOF-74, Fe-MOF-74 and Mg-MOF-74. The interactions with the IRMOFs show stronger interaction (in blue) between the hydrogen of 5FU and the oxygen from the inorganic unit. This interaction has an electrostatic character.

For M-MOF-74 it was observed that one of the oxygen atoms (O7 and O8) of 5FU coordinates to the metallic centre with a strong electrostatic character (intense blue colour) for Cu-MOF-74, Fe-MOF-74 (Fig. 11) and Mg-MOF-74. For Zn-MOF-74 this interaction is weaker, and with low electrostatic character, hydrogen bond formation similar to those formed in IRMOFs was also observed.

The most intense interaction between 5FU and M-MOF-74 may be associated with the more significant electrostatic character of the interaction between oxygen and metal, which combined with the smaller pore volume compared to IRMOFs, decreases the amount of adsorbed 5FU through stronger interactions.

The DFT-D3 interaction energies for a 5FU molecule when interacting with the inorganic unit region can be seen in Table 5. From these results, it is observed that IRMOFs have increased the interaction energy probably due to the cluster size, remembering that this unit is the same as all IRMOFs available. Zn-MOF-74 presents the smallest interaction energy of M-MOF-74 in agreement to the GCMC results. Otherwise, Mg-MOF-74, Fe-MOF-74, and Cu-MOF-74 present the highest values of interaction energy. In general, the interaction energies are almost within almost the same range, between 10.89 and 13.09 kcal mol^−1^ for GCMC and between 10.54 and 16.13 kcal mol^−1^ for B3LYP-D3, excepting for Fe-MOF-74.

**Table 5:** Interaction energy between 5FU and inorganic unit regions for all MOFs available. Obtained using B3LYP-D3/6-31G from small portion of the system 5FU@MOF from GCMC simulations

MOF	Interaction energy (kcal mol^−1^)
IRMOFs	12.93
Mg-MOF-74	16.13
Fe-MOF-74	28.32^a^
Cu-MOF-74	15.00^a^
Zn-MOF-74	10.54

a Calculated at magnetic ground state.

Finally, the results showed that the pore volume and the metallic centre in the inorganic unit are relevant factors in choosing an adsorption drug carrier system.

## Conclusions

GCMC simulations for the adsorption of 5FU on the different MOFs were evaluated. A key insight is in relation to the linear dependence of pore volume for the IRMOFs adsorption, in contrast to the M-MOF-74. It is probably strongly correlated to the efficient interaction of 5FU molecules with each other, and the interactions occur preferably between O7 or O8 with H10, H11 and H12 atoms from 5FU.

The interaction energies showed that 5FU strongest interactions are with M-MOF-74, and are governed mainly by van der Waals forces, although the contribution from Coulomb term is more intense than in the IRMOFs. Basically, the contribution by Coulomb comes from the interaction of the 5FU molecule with the metal atoms and with the oxygens of its coordination sphere. For the interaction between 5FU molecules, the contribution from Coulomb term was higher than that of van der Waals, in most cases. In general, this contributed to average interaction energy above 11.00 kcal mol^−1^, which is much higher than the energy between 5FU and IRMOFs (did not exceed 6.00 kcal mol^−1^).

Finally, 5FU exhibited noticeable interaction with M-MOF-74 when compared to all IRMOFs. For these structures, the metallic centre that constitutes the inorganic unit has a relevant influence on the average adsorption energy and the loading. On the other hand, the pore volume of IRMOFs proved to be another relevant and determining factor in the quantification.

## Conflicts of interest

There are no conflicts to declare.

## Supplementary Material
